# Ultrasound: A Useful Tool in the Diagnosis and Localization of Ulnar Neuropathy at the Elbow

**DOI:** 10.31486/toj.21.0002

**Published:** 2021

**Authors:** Amanda Honsvall Hoefler, Emily M. Miller, Yuka Kobayashi, Andrew W. Gottschalk

**Affiliations:** ^1^ Department of Family Medicine, Division of Sports Medicine, University of California–Los Angeles, Los Angeles, CA; ^2^ Department of Family Medicine and Sports Medicine, Oregon Health and Science University, Portland, OR; ^3^ Department of Orthopedics, Sports Medicine Institute, Ochsner Clinic Foundation, New Orleans, LA

## CASE PRESENTATION

An 18-year-old, right-hand-dominant, collegiate baseball pitcher presents for evaluation of right elbow and forearm pain for several months following an increase in pitching volume. He describes a progressively worsening aching pain and numbness along the ulnar side of the forearm that radiates into the ring and small fingers. The patient's symptoms are exacerbated with throwing and wake him from sleep at night. He recently noticed reduced pitch velocity and decreased grip strength. The season is about to end, and he asks if he should continue to play.

## BACKGROUND

Ulnar neuropathy at the elbow (UNE), also known as cubital tunnel syndrome, is the second most common compression neuropathy after carpal tunnel syndrome in both athletes and the general population.^1^ Patients present with vague discomfort localized to the medial elbow, paresthesia in the ring and small fingers, and weakness with pinching or gripping. Symptoms often worsen with activities requiring elbow flexion.^2^

In athletes, UNE may result from injury (blunt trauma or excessive stretching/traction), ulnar nerve instability, repetitive compression because of overuse, incorrect biomechanics or technique, and ill-fitting sports equipment. UNE has been well documented in overhead throwing athletes, most notably afflicting baseball players.^3-5^ The high incidence among overhead throwers is often secondary to medial elbow laxity because of enormous valgus stress during the acceleration phase of overhead throwing, resulting in compression and traction across the ulnar nerve.^6^

The ulnar nerve may become entrapped or compressed at various locations along the medial elbow and is a complicated region to evaluate (Table).^7-9^ Electrodiagnostic studies (EDx), including nerve conduction studies and electromyography (EMG), are the first-line diagnostic tool for UNE, with sensitivity of 37% to 86% and specificity of 95%.^10,11^ Downsides to EDx include low-variable sensitivity, increased risk of false negatives in early and late stages of UNE, invasiveness, and associated patient discomfort.^12,13^ Magnetic resonance imaging (MRI) is an alternate diagnostic tool with sensitivity of 90% and specificity of 80% for UNE, but MRI is limited by high cost and the inability to visualize the nerve along its entire length.^14,15^ In contrast, high-definition ultrasound has emerged as a powerful clinical diagnostic tool for the evaluation of compression mononeuropathies because of its ability to dynamically evaluate for nerve hypermobility or hypomobility, visualize anatomic variations, and identify pathology, in addition to its lower cost and greater patient convenience compared to MRI and EMG.^16-20^ On ultrasound, the compressed ulnar nerve appears swollen and hypoechoic (ie, dark compared to surrounding structures), with loss of its fasciculate appearance at or just proximal to the site of compression.^18^

**Table.** Anatomic Sites of Ulnar Nerve Entrapment and Compression at the Elbow, Listed Proximal to Distal

Anatomic Region	Compression Site
Cubital tunnel inlet (most common pathology)	Medial intermuscular septum Arcade of Struthers
Cubital tunnel	Osborne ligament Medial collateral ligament Anconeus epitrochlearis
Cubital tunnel outlet	Two heads of the flexor carpi ulnaris
Proximal forearm	Deep fascial bands of flexor carpi ulnaris Confluence of fascia from the flexor digitorum superficialis to the ring finger

## REVIEW OF EVIDENCE

Studies have demonstrated a strong correlation between the location of ulnar nerve slowing on EDx and ulnar nerve enlargement or increased ulnar nerve cross-sectional area (UNCSA) on ultrasound.^15,18,21^ A 2017 study by Podnar et al sought to compare EDx and ultrasound for the precise localization of UNE in 160 patients.^17^ They expected to find a strong correlation between ulnar nerve constriction and slowing, but instead they found the highest degree of motor nerve slowing at the location where the nerve was most enlarged.^17^ This correlation was true for compression at the retrocondylar groove (n=106) and for entrapment under the humeroulnar aponeurotic arcade (n=54). For entrapment under the humeroulnar aponeurotic arcade, motor nerve slowing and increased UNCSA were most pronounced just proximal to the location of nerve entrapment.^17^

Increased UNCSA measured by ultrasound has been shown to have a high sensitivity and specificity for diagnosis of UNE, although specific cutoff values and measurement locations have varied. An important note is that although severity of UNE can be defined by electrophysiologic measures using the preoperative aspects and dimensions used for an anatomical (PADUA) classification, classification by symptom severity has not been established for UNE, making it difficult to discuss patient populations with uniformity.^22^ A 2012 study by Ayromlou et al demonstrated that UNCSA was significantly greater in patients with UNE (n=29) vs healthy controls (n=35) (*P*<0.001).^15^ UNCSA also differed significantly between UNE severity grades based on EDx (*P*<0.05), with the maximum UNCSA (CSA-max) showing the greatest sensitivity (93%) and specificity (68%) for UNE diagnosis at a cutoff value of >6 mm^2^.^15^ Other studies have reported CSA-max cutoff values of 8.3 mm^2^ to 11 mm^2^, yielding sensitivities of 88% to 100% and specificities of 88% to 98% for UNE diagnosis.^22-25^ More recently, a 2019 study by Rayegani et al demonstrated that UNCSA was significantly larger in the symptomatic UNE group (n=32) than in the asymptomatic control group (n=34), with the greatest ultrasound diagnostic value measured 2 cm distal to the medial epicondyle (ME) and a cutoff value of 9 mm^2^ (sensitivity 84% and specificity 80%).^26^ Notably, UNCSA at the ME was significantly larger in UNE with greater severity based on EDx (*P*=0.006), with longer duration of symptoms (*P*=0.0001), and with predominant axonal loss (P<0.01).^26^ Further, a 2018 study by Terayama et al found that UNCSA measured by ultrasound was significantly larger in the UNE group (n=12) than in healthy controls (n=24), with high interrater and intrarater reliabilities.^27^ They found UNCSA was maximal at 1 cm proximal to the ME, where UNE could be discriminated by a cutoff value of 11.0 mm^2^ (sensitivity 92%, specificity 90%) and an area under the receiver operating characteristic curve of 0.96.^27^

New evidence suggests that ultrasound may offer greater sensitivity than EDx in early and mild disease. In a 2015 study by van Veen et al comparing the 2 modalities in 30 UNE cases and 33 healthy controls, ultrasound was more sensitive than EDx (76.7% vs 63.3%, respectively) but less specific (72.7% vs 87.9%, respectively) for diagnosing UNE.^20^ van Veen et al also found that ultrasound was more sensitive for diagnosis of UNE if symptoms were present for ≤6 months, whereas EDx had greater sensitivity and specificity for symptom duration >6 months.^20^ A 2019 study by Pelosi and Mulroy found that ultrasound had higher sensitivity than EDx in detecting clinically very mild (20% vs 3%, respectively) and mild (62% vs 47%, respectively) UNE.^28^ Ultrasound measurement of UNCSA had a strong positive correlation with both clinical and EDx severity scores but with significant overlap across the severity groups as reported in many studies.^11,15,17,26,28-30^

Ultrasound can also offer significant value in evaluation of UNE with abnormal nonlocalizing electrophysiology (NL-UN) on EDx.^31^ A 2018 study by Pelosi et al assessed demographic, clinical, EDx, and ultrasound characteristics of NL-UN.^32^ They found that 25% of EDx-proven UNE had NL-UN (n=16) with moderate or severe clinical and electrophysiological ratings.^32^ Ultrasound was able to localize focal UNE in 13 of the 16 NL-UN cases, diffuse ulnar nerve abnormality in 3 cases, and a likely or possible causative mechanism in 11 cases.^32^ In another 2018 study, Alrajeh and Preston found that 12 of 56 patients with UNE had NL-UN on EDx.^31^ Ultrasound was able to localize the site of pathology in 100% of these cases, allowing for more comprehensive evaluation and precise surgical planning than EDx for these electrically nonlocalizable lesions.^31^

## TAKEAWAY

High-resolution ultrasound is a sensitive and specific diagnostic tool for identification of UNE. Many studies have demonstrated remarkable consistency between ultrasound and EDx for UNE diagnosis. Ultrasound appears to be superior to EDx in localization of early, mild, and electrically nonlocalizable UNE. However, the current body of literature is limited by small sample sizes, heterogeneity in patient selection, and variability in measurement parameters.

Ultrasound is a valuable clinical tool, allowing for earlier and more precise localization of UNE, in addition to dynamic visualization of pertinent anatomy such as nerve hypermobility, lesions, and anatomic variations that may impact interventional approach. Early identification and management of UNE in athletes is associated with improved outcomes, as untreated neuropathies have the potential to progress from paresthesia to paralysis and loss of intrinsic muscles.^1^ In general, nonoperative management with rest, anti-inflammatory medications, nighttime splinting for up to 6 weeks, and physical therapy with a gradual interval-throwing program and posterior capsule stretching program have been shown to be successful.^6^ In patients with refractory or recurrent UNE, ulnar nerve decompression with or without transposition can lead to excellent outcomes.^6^

## CASE RESOLUTION

This baseball pitcher's history and examination were concerning for UNE. EDx was negative. High-resolution ultrasound performed by an experienced sports medicine physician identified compression of the ulnar nerve within the cubital tunnel by the Osborne ligament, with increased UNCSA of 10 mm^2^ measured 2 cm distal to the ME. Following a failed trial of conservative management, the patient underwent ulnar nerve decompression with transposition. Of note, the area of compression on ultrasound correlated to intraoperative findings. The patient was able to successfully return to pitching following a graduated throwing progression and a posterior capsule stretching program.

## Notes

This article meets the Accreditation Council for Graduate Medical Education and the American Board of Medical Specialties Maintenance of Certification competencies for Patient Care and Medical Knowledge.
